# The c-Jun N-terminal kinase prevents oxidative stress induced by UV and thermal stresses in corals and human cells

**DOI:** 10.1038/srep45713

**Published:** 2017-04-04

**Authors:** Lucile Courtial, Vincent Picco, Renaud Grover, Yann Cormerais, Cécile Rottier, Antoine Labbe, Gilles Pagès, Christine Ferrier-Pagès

**Affiliations:** 1 Centre Scientifique de Monaco, Marine Department, 8 Quai Antoine Ier, MC-98000 Monaco, Principality of Monaco; 2 Sorbonne Universités, UPMC Univ Paris 06 IFD-ED 129, 4 Place Jussieu, 75252 PARIS cedex 05, France; 3 UMR ENTROPIE (IRD, Université de La Réunion, CNRS), Laboratoire d’Excellence « CORAIL», BP A5, 98848, Nouméa cedex, New Caledonia; 4 Centre Scientifique de Monaco, Biomedical Department, 8 Quai Antoine Ier, MC-98000 Monaco, Principality of Monaco; 5 University Nice Sophia-Antipolis, Institute for Research on Cancer and Aging of Nice (IRCAN), CNRS UMR7284/INSERM U1081, Centre Antoine Lacassagne, Nice 06189, France

## Abstract

Coral reefs are of major ecological and socio-economic interest. They are threatened by global warming and natural pressures such as solar ultraviolet radiation. While great efforts have been made to understand the physiological response of corals to these stresses, the signalling pathways involved in the immediate cellular response exhibited by corals remain largely unknown. Here, we demonstrate that c-Jun N-terminal kinase (JNK) activation is involved in the early response of corals to thermal and UV stress. Furthermore, we found that JNK activity is required to repress stress-induced reactive oxygen species (ROS) accumulation in both the coral *Stylophora pistillata* and human skin cells. We also show that inhibiting JNK activation under stress conditions leads to ROS accumulation, subsequent coral bleaching and cell death. Taken together, our results suggest that an ancestral response, involving the JNK pathway, is remarkably conserved from corals to human, protecting cells from the adverse environmental effects.

Living organisms have developed adaptive mechanisms allowing them to survive in a constantly changing environment^1^,^2^. Organisms that can rapidly adjust to these changes are likely to exhibit a greater degree of adaptation to a variety of stresses^3^. At the molecular level, cells have thus implemented complex signalling pathways to sense danger, and trigger a sequence of events that will mitigate any adverse effects. Among these pathways, mitogen-activated protein kinases (MAPKs) stimulate the expression of genes involved in the response to stress^4^. MAPKs also control key cellular processes such as proliferation, differentiation, migration, survival and apoptosis^5^. The MAPK gene family encompasses three major subgroups: the extracellular signal regulated kinase (ERK), the p38/HOG and the c-Jun N-terminal kinase (JNK) groups. JNKs are evolutionarily conserved and are found in all eukaryotes, including plants, vertebrates and anthozoans^6^,^7^. When activated by dual phosphorylation of a threonine-proline-tyrosine (TPY) motif, this family of kinases plays a key role in the response to thermal and ultra-violet radiation (UVR) stresses in animals as well as in several terrestrial and marine plants^8^,^9^,^10^,^11^,^12^,^13^.

In human skin cells, which are often exposed to high solar radiation, UVR-induced reactive oxygen species (ROS) have been suggested to induce JNK activation^14^, leading in turn to upregulation of anti-oxidant enzymes to overcome ROS accumulation^4^,^13^,^15^. Moreover, in cells, JNK has been proposed to have both pro-survival and pro-apoptotic activities, depending on the duration and intensity of the stress as well as the cellular context being examined^16^,^17^. Due to these different outcomes following activation of the JNK pathway, the primary function of JNK in the early response to UVR and thermal stress is still unclear^16^,^18^,^19^,^20^.

Tropical corals are also exposed to high solar UVR and high temperature levels, especially in the face of climate change, which have already led to more than 20% destruction of coral reefs^21^,^22^. Exposure to these stresses leads to coral bleaching, i.e. the loss of coral algal symbionts and/or photosynthetic pigments^23^,^24^, resulting in a drop in photosynthesis and calcification^25^, and possibly leading to coral death. At the cellular level, bleaching has been proposed to be the consequence of an elevated production of ROS which damages cell components^26^. To prevent or to fight oxidative stress, corals have implemented defence strategies such as the synthesis of fluorescent pigments, heat-shock proteins, mycosporin-like amino acids^27^,^28^ and the production of anti-oxidant enzymes^26^. An increasing number of studies indicate that corals express genes encoding MAPKs, including those encoding JNK^29^,^30^,^31^,^32^,^33^,^34^. This suggests the presence of a conserved stress response between coral and human cells at the molecular level, although the phylum cnidaria diverged from bilateria 550 million years ago^35^. An increase of the expression of genes coding for canonical substrates of JNK (c-Jun and c-Fos) was indeed observed in the coral *Acropora sp*. under thermal stress and following wounding^34^,^36^,^37^. Such induction may be specific to corals since post-translational and not transcriptional regulation of JNK signalling is most often observed in mammalian cells^38^. Although the above studies suggest a conservation of the stress response involving MAPK signalling cascades in corals, their specific functions have not been clearly defined. In particular, MAPK signalling pathway activity, which is classically considered the most appropriate readout, has never been monitored in corals.

Taking advantage of the amino acid sequence conservation of coral and human JNK orthologs, in particular the phosphorylatable TPY motif contained in the kinase domain, we used commercially available antibodies to analyse JNK activation upon thermal and UVR stress in both corals and human skin fibroblasts. Our aims were to confirm the presence of the JNK protein in corals, to study its phosphorylation (i.e. activation) under thermal and UVR stress, and to compare JNK activation in corals and human fibroblasts. In addition, we also endeavoured to link the JNK pathway activation to the defence against stress-induced ROS production in both models.

## Results

### Analysis of anthozoan transcriptomes

**Figure 1:**
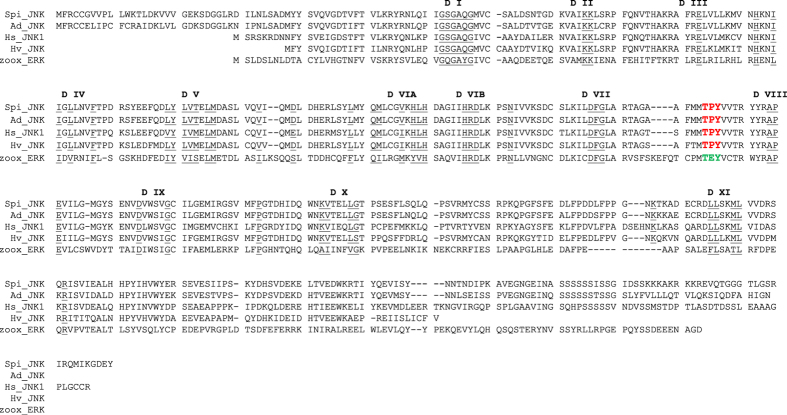
Sequence alignment of MAPK orthologs. The JNK orthologs of *Stylphora pistillata* (Spi-JNK), *Acropora digitifera* (Ad-JNK), *Hydra vulgaris* (Hv-JNK), the human MAPK8/JNK1 (Hs-JNK1) and the *Symbiodinium sp*. ERK ortholog (zoox_ERK) protein sequences are shown. The JNK-specific TPY activation motif and the ERK-specific TEY motif are highlighted in red and green respectively. The eleven conserved kinase domains are underlined.

The human protein sequence for MAPK8/JNK1 (NCBI reference sequence: NM_001323322.1) was blasted against sequence databases including *Stylophora pistillata*^39^ (GENBANK: GARY01004588), *Acropora digitifera*^40^ (NCBI reference sequence: XM_015907276), *Hydra vulgaris*^41^ (NCBI reference sequence: NM_001309762) and the coral symbiotic unicellular algae *Symbiodinium* clade B1^42^ (accession number symbB.v1.2.036439). A sequence showing 89%, 74% and 64% homology with the protein sequences of *A. digitifera* (Ad-JNK), *H. vulgaris* (Hv-JNK) and the human MAPK8/JNK1 (Hs-JNK1), respectively, was identified in *S. pistillata*. This sequence (hereafter referred to as Spi-JNK for *S. pistillata* JNK) contains both the conserved kinase domains^43^ and the TPY motif unique for JNK orthologs^15^, like Ad-JNK, Hv-JNK and Hs-JNK sequences (Fig. 1). These results suggest that a single ortholog of JNK is present in *S. pistillata*, as opposed to the three genes encoding several JNK isoforms in the human genome^44^. Note that the unique and closest ortholog present in the *Symbiodinium* sequence database showed 35% homology with Spi-JNK (Fig. 1) and contained a TEY motif characteristic of ERK, instead of a TPY motif.

The TPY domain of JNK is phosphorylated by kinases upstream in its signal transduction pathway and as such its phosphorylation is an established readout of JNK activation. We therefore hypothesized that Spi-JNK, like its human counterpart, is activated by phosphorylation of the threonine and tyrosine residues contained in the TPY motif. To investigate this, we used commercially available anti-human JNK antibodies to test whether phosphorylated JNK (pJNK) could be detected in *S. pistillata* extracts. We used human fibroblasts to validate our approach and took advantage of the absence of a JNK ortholog in the *Symbiodinium* genome (Fig. 1) to prevent any misinterpretation due to potential cross contamination of animal and algae proteins in total protein extracts.

### Characterization of JNK activation in corals and human cells

**Figure 2:**
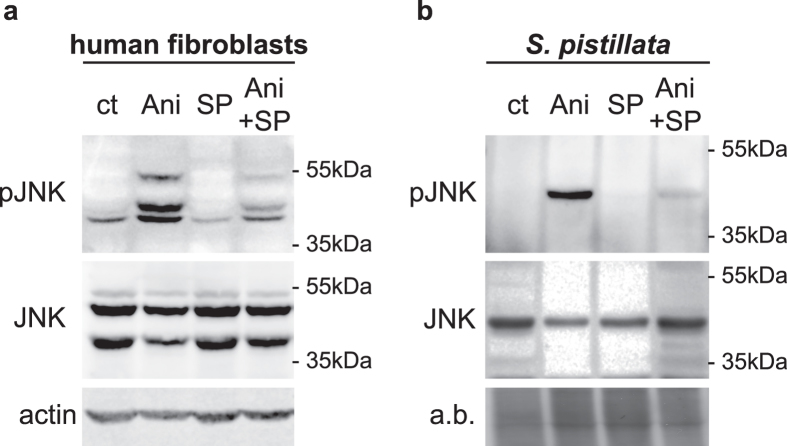
Detection of JNK activation in human fibroblasts and corals. Immunoblot revealing activated (pJNK) and total forms of JNK (JNK) present in (**a**) human fibroblasts and (**b**) *S. pistillata* nubbins incubated in the absence (control; ct) or presence of SP600125 (SP), anisomycin (Ani), or combination of both (Ani + SP). Molecular weight standards in kilodaltons (kDa) are indicated on the right side of the blots. (**a**) Total JNK and actin served as loading controls for human fibroblasts. (**b**) Total JNK and amido black staining (**a**,**b**) served as loading controls for *S. pistillata*. Data are representative of at least three independent experiments.

In corals, in agreement with the transcriptome analysis, only one JNK protein was present and therefore expressed in the western blots (Fig. 2a), while, in human fibroblasts, only JNK1 and JNK2 proteins were expressed (demonstrated by siRNA knockdown experiments, Fig. 2b and Fig. S1). In control culture conditions, phosphorylated forms of JNK (pJNK) were barely detectable in fibroblasts and undetectable in coral extracts (Fig. 2a and b). However, anisomycin, an activator of JNK^45^, induced significant phosphorylation of JNK in both fibroblasts and *S. pistillata* (Fig. 2a and b). In fibroblasts, anisomycin induced the phosphorylation of isoforms that are not detected in the control condition.The anti-human pJNK antibody detected a unique immuno-reactive band in coral extracts, suggesting that this antibody recognized the phosphorylated TPY motif present in Spi-JNK (Fig. 2b). Consistent with our results indicating the absence of a JNK gene in the symbiont genome, isolated symbiont extracts did not present any immuno-reactive band when probed with the pJNK antibody (Supplementary Fig. S2). To further determine the specificity of the anti-human pJNK, we used the pharmacological inhibitor SP600125, commonly used as a JNK inhibitor^46^,^47^. It prevents autophosphorylation of JNK through competition with ATP, and the subsequent phosphorylation of JNK targets^48^. Treatment with SP600125 reduced the basal pJNK level in fibroblasts (Fig. 2a) and strongly reduced anisomycin induced of JNK phosphorylation in both *S. pistillata* and fibroblasts (Fig. 2a and b).

### Both Spi- and Hs-JNK are activated by a combined thermal and UVR stress

**Figure 3:**
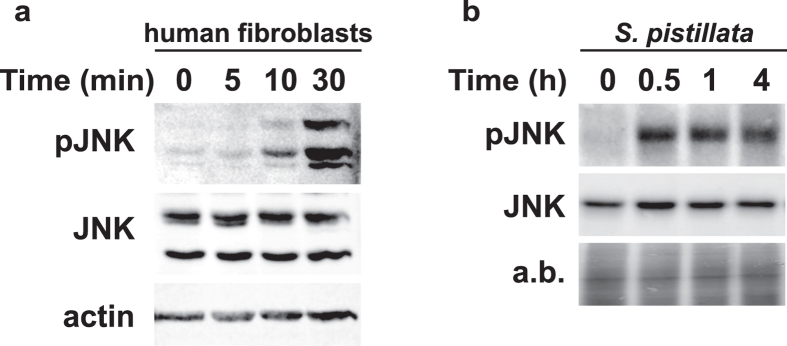
Time course of JNK activation by combined UVR and thermal stresses in human fibroblasts and coral. Immunoblot revealing activated (pJNK) and total JNK (JNK) in (**a**) human fibroblasts or (**b**) *S. pistillata* nubbins exposed to UVR and temperature rise for the indicated times. (**a**) Total JNK and actin served as loading controls for human fibroblasts. (**b**) Total JNK and amido black staining (**a**,**b**) served as loading controls for *S. pistillata*. Data are representative of three independent experiments.

To monitor the kinetics of JNK activation upon modifications of culture conditions, we subjected fibroblasts and coral nubbins to a combination of heat shock and UVR stress. In human cells, JNK activation was detectable after 10 minutes and reached a plateau after 30 minutes exposure in this experimental condition (Fig. 3a). In corals, JNK activation was at its maximum within 30 minutes and these levels were maintained at least for up to 4 hours under stress condition (Fig. 3b). Hereby, we show that, like in human cells, JNK activation is involved in the early response of corals to stress.

### Respective contributions of UV and thermal stress to JNK activation

**Figure 4:**
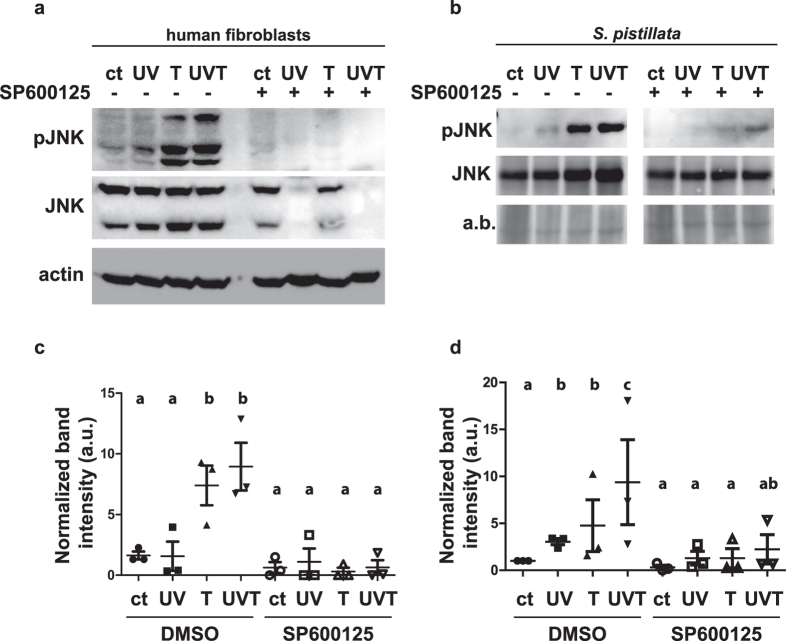
JNK activation by UVR, temperature rise and combined UVR and temperature rise in human fibroblasts and coral. Immunoblot revealing activated (pJNK) and total JNK (JNK) present in (**a**) human fibroblasts and (**b**) *S. pistillata* exposed to UVR (UV), increased temperature (T) or the combination of both stresses (UVT) in the absence (−) or presence (+) of SP600125. Total JNK and actin served as loading controls for human fibroblasts; total JNK and amido black staining (**a**,**b**) served as loading controls for *S. pistillata*. Data are representative of three independent experiments. Densitometric analysis of activated JNK intensities was performed on three independent blots for (**c**) human fibroblasts and (**d**) *S. pistillata*. The medians and standard deviations of three independent experiments are represented. Statistically significant differences, from the Tukey’s post hoc test are indicated with letters. Similar letters indicates no significant differences between conditions.

The effects of UV and temperature on early JNK activation were tested by subjecting fibroblasts and corals to the individual and combined stresses for 10 and 30 minutes, respectively. Ten minutes exposure to UVR alone had no significant effect on JNK activation in fibroblasts (Fig. 4a,c and Supplementary Table 1), although a longer exposure induced a strong activation (Supplementary Fig. S3). On the contrary, exposure to UVR triggered a detectable JNK phosphorylation in corals (p = 1.4 × 10^−3^) (Fig. 4b,d and Supplementary Table 1). Thermal stress strongly induced JNK activation in both models (p = 1.25 × 10^−3^ and p = 5.38 × 10^−3^ respectively). The combination of UV and thermal stress resulted in slightly higher but not statistically significant activation of JNK as compared to thermal stress alone (Fig. 4b,d and Supplementary Table 1). Treatment of cells and corals with the JNK inhibitor strongly reduced stress-induced JNK activation (p = 6.62 × 10^−5^ and p = 6.51 × 10^−5^ respectively). Notably, combining JNK inhibition to UVR stress had a dramatic effect on JNK protein stability in fibroblasts (Fig. 4a). We performed experiments to rule out the possibility that cell membrane disruption led to a leakage of the proteins out of the cells. First, we showed that the ROS carboxy-DCFDA probe used for ROS quantification did not leak out of the cells even after more than 30 minutes of exposure to a combination of UVR and SP600125 (Supplementary Fig. S4a). Moreover, the quantification of the staining of the cells with the non-permeant fluorescent dye propidium iodide (PI) showed no difference between the control and UV + SP conditions (Supplementary Fig. S4b). These results suggest that the disappearance of the JNK bands was not associated to cell membrane disruption. We then hypothesized that JNK degradation could be due to a cleavage by proteases. We first showed that JNK degradation is not caused by caspase cleavage as caspase inhibitor Z-VAD-FMK did not prevent the degradation of JNK protein in cells exposed to a combination of UV and SP600125 (Fig. S5). Moreover, western blot analysis of the PARP protein showed a cleavage of PARP in the UV + SP condition (Fig. S5). Interestingly, this cleavage was not inhibited by Z-VAD-FMK and resulted in a band of different size than the staurosporin-induced caspase-dependent one, suggesting that non-caspase proteases are activated in these conditions. To test a potential autophagy-driven degradation of JNK, we assessed the expression level of the SQSTM1 protein. We observed no increase in SQSTM1 expression level, suggesting that the UV + SP treatment did not activate autophagy (Fig. S5). Finally, treatment with the proteasome pharmacological inhibitor MG132 did not result in stabilization of JNK in the UV + SP condition, suggesting that JNK is not degraded by the proteasome either (Fig. S6).

### Inhibition of JNK in stress conditions impairs ROS scavenging capacities and results in strong adverse effects in corals and human cells

**Figure 5:**
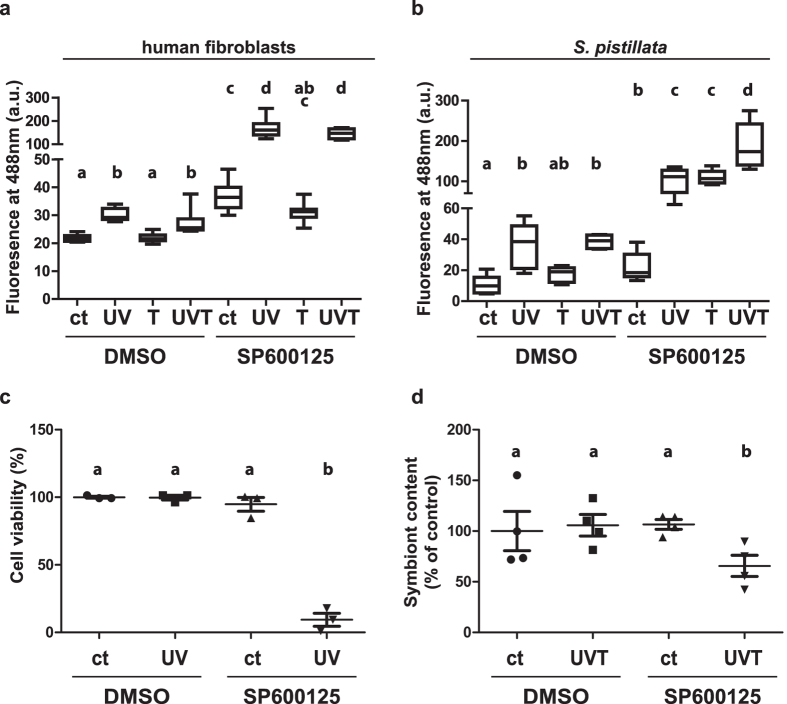
JNK activity protects corals and human cells from UV and temperature-induced oxidative stress and their adverse effects. Staining intensity of carboxy-H_2_DCFDA (fluorescence at 488 nm), indicative of the presence of ROS, evaluated after 10 and 30 minutes respectively for (**a**) human fibroblasts and (**b**) *S. pistillata* with or without pre-incubation with SP 600125 in the following conditions: control (ct), UVR (UV), high temperature (T) and a combination of UVR and high temperature (UVT). The median, standard deviation and 95% confidence interval of at least three independent experiments are represented as boxplots. (**c**) Cell viability of human fibroblasts treated or not with SP600125 for 30 minutes prior to exposure to 10 minutes control (ct) or UVR (UV) measured 24 hours after the beginning of the experiment. (**d**) Symbiont content of *S. pistillata* nubbins treated or not with SP600125 for 1 hour prior to exposure to 4 hours control (ct) or combined UVR and thermal stresses (UVT) measured 24 hours after the beginning of the experiment. Medians and standard deviations are represented. Statistically significant differences, from the Tukey’s post hoc test are indicated with letters. Similar letters indicates no significant differences between conditions.

To gain more insight into the role of JNK activation in the early stress response, we assessed ROS levels in the experimental conditions described above. In both fibroblasts and corals, UVR induced a significant increase in ROS concentration (p < 2 × 10^−16^ and p = 1.78 × 10^−10^ respectively) (Fig. 5a,b and Supplementary Table 2). However, in both systems, thermal stress alone had no or very low effect on ROS content in the absence of JNK inhibitor (Fig. 5a,b and Supplementary Table 2). In corals, the inhibition of JNK activity leaded to an increase in ROS concentration in all conditions (p = 4.60 × 10^−13^) (Fig. 5b and Supplementary Table 2). A significant interaction between UVR and temperature led to a higher ROS concentration compared to each stress alone (p = 2.53 × 10^−3^) (Fig. 5b and Supplementary Table 2). In fibroblasts treated with JNK inhibitor, the ROS concentration did not change under high temperature but strongly increased under UVR with or without high temperature (p < 2 × 10–16) (Fig. 5a and Supplementary Table 2).

Next we studied the physiological consequences of increased ROS cellular concentrations in human fibroblasts and corals. Hence, we determined the fibroblast viability and the loss of symbiont (bleaching) in coral as read-outs of ROS-mediated stress. As combined thermal stress and JNK inhibition had no effect on ROS content in fibroblasts, we only assessed the UVR response. Exposure to UVR or to SP600125 alone did not affect the fibroblasts viability, while the mean cell survival upon exposure to UVR plus the JNK inhibitor was less than 10% (p = 1.07 × 10^−3^) (Fig. 5c and Supplementary Table 3). In corals, exposure to SP600125 or UVT alone did not induce bleaching. However, exposure to the combination of UVR and high temperature in the presence of the JNK inhibitor for 24 hours led to a loss of more than 30% of the symbionts (p = 1.21 × 10^−2^) (Fig. 5d and Supplementary Table 3). These results suggest that JNK activity is necessary for the organisms to control the advert effects triggered by the stress-induced ROS burst.

## Discussion

While the physiological response of corals to UVR or thermal stress has been well studied^27^,^28^, the intracellular signalling that allows corals to rapidly respond to these stresses is still unknown. Our results strongly suggest the presence of a unique JNK protein in corals. This is in agreement with what was found in other cnidarians such as *Nematostela vectensis* or *Hydra vulgaris*^49^,^50^. For the first time, we showed a 100% conservation of the TPY motif in the catalytic domain of Spi and Hs-JNK. Phosphorylation of this domain by upstream kinases is essential for JNK activation. Hence, specific antibodies directed against the phosphorylated forms of Hs-JNK, which is an established player in the mammalian stress response^15^, have been generated to monitor Hs-JNK activation. The specificity of these antibodies was further demonstrated for corals, since, as in human cells, Spi-JNK was activated and/or inhibited by the same mammalian activator/inhibitor. In addition, a significant increase in JNK phosphorylation was observed under exposure to high temperature and/or UVR, suggesting that activation of this pathway is a common response of *S. pistillata* to various environmental disturbances. Although previous studies suggested that mRNA encoding members of the JNK signalling pathway could be up-regulated in the corals’ response to chronic stress conditions^34^,^36^,^37^, JNK protein activity, which is classically considered the most appropriate readout, especially in an acute response setting, has never been monitored in corals. Our study thus demonstrates that JNK activity in corals is stimulated by environmental conditions in a way comparable to mammalian cells^4^,^51^.

In *S. pistillata*, the phosphorylation of JNK was higher under thermal stress than UVR, and even seemed to be higher under the combination of both stressors, suggesting a cumulative effect of these two variables. This cellular response is also in agreement with the corals’ physiological response to the same conditions^25^,^52^,^53^ (Courtial *et al. in press*). Corals are usually well protected from UVR by a panel of natural sunscreens, such as mycosporine like amino acids^54^ whereas they are more sensitive to thermal stress alone or in combination with other stressors^55^. Phosphorylation of JNK under the same stresses was previously observed in two kelp algae, but their response was mostly driven by UVR^11^. Nonetheless, a cumulative effect of temperature and UVR on JNK activation was observed in algae, suggesting that JNK is a good proxy for stress in marine organisms. Its activation is also proportional to the intensity of the stress. Similar to corals and algae, increased phosphorylation of JNK was observed in human fibroblasts under thermal stress. Instead, the UV stress inflicted to human cells was below the threshold value necessary to enhance the basal JNK activity (Fig. 4 and Supplementary Fig. S3). Nonetheless, the combination of UVR and JNK inhibition significantly decreased fibroblast viability, suggesting that the basal JNK activity is necessary and sufficient to overcome a moderate UV stress. Moreover, we observed a decrease in total JNK amount in fibroblasts by combining UVR and JNK inhibition. Such rapid disappearance of JNK has never been described and was not associated to cell membrane disruption, to caspase cleavage, to autophagy processes or to proteasomal degradation. Although we don’t have a definitive explanation for such disappearance, the caspase-independent cleavage of PARP suggests that other proteases are activated in these conditions and may also cleave JNK^56^. In both corals and fibroblasts, JNK inhibition by SP600125 indeed resulted in ROS accumulation. This “binary result” strongly suggests that JNK plays a pivotal role in protecting the cells against ROS damages; however, based on our results, it is difficult to conclude whether JNK activation results from ROS production or from another mechanism. In fibroblasts, the inactivation of JNK by SP600125 during exposure to fairly low dose of UVR^57^,^58^ (3 W.m^−2^ UVB and 30 W.m^−2^ UVA for 10 minutes) led to the death of human cells. This observation supports the concept that, in the absence of active JNK, the fibroblasts cannot tolerate and neutralized the excessive production of UVR-induced ROS. This result contrasts with some studies showing that JNK activity is necessary for UVR-induced apoptosis^14^,^59^,^60^. However, our results are consistent with studies suggesting that JNK is also a key modulator of oxidative stress-mediated cell survival^14^,^60^,^61^,^62^. Discrepancies between studies may be explained by the use of different UV sources: whereas a UVA/B source that reproduces the UVR solar spectrum was used in our study, cells were exposed to UVC in the aforementioned studies^14^,^59^,^60^. In corals, the inactivation of the JNK pathway led to a slight bleaching (loss of symbiont cells) under the combined UVR/thermal stress, supporting the antioxidant function of the JNK signal transduction pathway. Consistent with our results, a protective role of JNK towards oxidative stress has also been reported in *C. elegans* and *D. melanogaster*^63^,^64^,^65^,^66^, suggesting that this function is one of the primary functions of JNK throughout the animal kingdom. In humans, JNK activation has been linked to the upregulation of Manganese dependent-Super Oxide Dismutase (Mn-SOD) and catalases depending on the deacetylase SIRT1 and the FOXO transcription factor^13^,^64^. In a more recent study, the early JNK response has been proposed to inhibit apoptosis^67^. Collectively, the results obtained in this study and other studies on various organisms suggest that the primary role of JNK signalling in thermal and solar spectrum UVR-induced oxidative stress consists of triggering a pro-survival anti-oxidant response. However, in case of a longer or more potent stress, e.g. exposure to UVC radiation^59^, JNK might play a role in the induction of apoptosis.

Overall, our results suggest that JNK activation may play an evolutionarily-conserved role from cnidarians to mammals in protecting cells from oxidative stress. As the intensity of JNK phosphorylation is directly related to the intensity of oxidative stress in corals, JNK activation may represent a good and rapid proxy and an easily detectable marker in field studies to predict the effects of environmental perturbations on corals. In a time of rapid climate change, the knowledge of cellular pathways involved in the response of corals to stress is particularly important. Our findings bring new insight into the mechanisms that corals employ to face environmental pressures and raise several questions about the functions of JNK in both coral and human cells.

## Methods

### Maintenance of coral nubbins and human fibroblasts

The scleractinian coral *Stylophora pistillata (Pocilloporidae* family) was chosen as a model because it has a broad geographic range (from the Red Sea to the western central Pacific^68^ and is widely used in laboratory experiments involving environmental stress effects, including short term thermal stress and UV exposure^25^. Colonies collected in the Gulf of Aqaba (Red Sea, Jordan) were cultured as previously described in Ferrier pages *et al*.^25^.

Skin fibroblasts (BJ-EHLT cells) were kindly provided by E. Gilson’s lab (IRCAN) and cultured in Dulbecco’s Modified Eagle’s Medium (DMEM) (Invitrogen, Villebon-sur-Yvette, France) supplemented with 10% heat-inactivated fetal calf serum (FCS, Dutscher, Brumath, France) at 37 °C in an atmosphere of 5% CO_2_ as previously described^69^.

### Antibody validation

The antibody directed against the activated JNK (pTPpY) was from Promega, Madison, Wisconsin (V7931); the pan-JNK antibody was from R&D Systems, Minneapolis, Minnesota (AF1387).

Coral nubbins were incubated in individual beakers filled with 40 mL of 0.45 μm filtered seawater and placed during one hour under the following conditions: 1) 0.005% DMSO (drug solvent), 2) 100 μmol.L^−1^ anisomycin, which is a JNK activator^70^ (Euromedex, Souffelweyersheim, France), 3) 40 μmol.L^−1^ of SP600125, which is a JNK inhibitor^48^ (Euromedex); 4) a condition in which nubbins were pre-incubated with SP600125 during one hour, then stimulated with anisomycin while kept in the presence of SP600125 for another hour. The incubation medium was continuously stirred using magnetic stirrers at a constant temperature of 25 °C and a photosynthetically active radiation of 200 μmoles photons m^−2^ s^−1^. At the end of the incubation, nubbins were frozen and kept at −80 °C prior to western blot analysis.

Skin fibroblasts (BJ-EHLT cells) were seeded at a density of 2 × 10^6^ cells in 60 mm diameter dishes, and were subjected to the same treatments as the coral nubbins 24 hours later.

A *Symbiodinium* sp. culture (clade A1 isolated from *S. pistillata*,) in ASP8 medium^71^ at 25 °C and 150 μmoles photons m^−2^ s^−1^ was also used to check for the presence of the JNK protein. Samples were exposed to the same 4 conditions as the coral nubbins, pelleted by centrifugation at 14 g for 10 minutes, frozen and kept at −80 °C prior to western blot analysis.

### Assessment of JNK activity in response to combined thermal and UV stress

High temperature and UVR stresses were applied to corals and human cells. For corals, the thermal stress corresponded to an increase in temperature from the normal culture condition of 25 °C to 30 °C. The UVR stress corresponded to an increase in UVR from 0 (HQI lamps in the culture conditions) to a radiation intensity of about 3 W.m^−2^ UVB and 30 W.m^−2^ UVA (Q-Panel UVA 340 lamps). These intensities correspond to those reaching the sea surface in tropical areas^25^. Similar dose were used in previous work on corals^72^,^73^,^74^ and in dermatological studies^57^,^58^. The UVR intensity was measured using ILT1400 portable radiometer and detectors (SEL240/UVB-1/TD and SEL033/UVA/TD) according to Shick *et al*.^75^. For human fibroblasts, thermal stress was induced by an increase in temperature from 37 °C to 42 °C, and UVR stress was the same as for coral nubbins.

JNK induction kinetics were evaluated on twelve coral nubbins, which were collected from the culture tanks and incubated during 4 h with constant agitation at 30 °C under UVR in beakers containing 40 mL of 0.45 μm filtered seawater. Three nubbins were frozen for each time point (0, 0.5, 1 and 4 hours). Fibroblasts were lyzed in Laemmli buffer prior to stress and after 5, 10 and 30 minutes incubations in the culture medium at 42 °C under UVR.

To assess JNK activity in response to individual and combined stress, coral nubbins were incubated in individual beakers, in the dark at 25 °C for 30 minutes in 40 mL of 0.45 μm filtered seawater containing 0.004% DMSO or 40 μmol.L^−1^ of JNK inhibitor (SP600125) prior to a 30 minutes exposure to the following conditions: 25 °C without UVR, 25 °C with UVR, 30 °C without UVR and 30 °C with UVR. Nubbins were flash frozen in liquid nitrogen and conserved at −80 °C prior to western blot analysis.

Similarly, BJ-EHLT fibroblasts were seeded at a density of 2 × 10^6^ cells in 60 mm diameter dishes 24 h before the experiment. The cells were then incubated with 0.001% DMSO or 10 μmol.L^−1^ of SP600125 for 30 minutes at 37 °C prior to a 10 minutes exposure to the following conditions: 37 °C without UVR, 37 °C with UVR, 42 °C without UVR and 42 °C with UVR.

### Western blot analysis

For coral samples, the tissue of each nubbin was removed from the skeleton using an air-pick in 1 mL of Laemmli buffer^76^. Samples were then sonicated for 30 seconds and centrifuged three times (5 minutes at 15 000 g) to remove the debris. Isolated *Symbiodinium* extracts were prepared from pellets re-suspended in 50 μL Laemmli buffer and then treated as the coral samples. Fibroblasts were washed twice in phosphate buffered saline (PBS), lyzed in Laemmli buffer directly in the dishes and sonicated for 30 seconds. For all samples, the total protein concentration was determined using a BCA protein assay kit (Thermo Fisher Scientific) according to the manufacturer’s recommendation. 1,4 Dithiothreitol (1 mM)-bromophenol blue solution (0.1% bromophenol blue) was added to all samples and they were then heated for 5 minutes at 95 °C and 60 μg of total protein were separated on 10% polyacrylamide gels and transferred onto a PVDF membrane at an intensity of 200 mA at 4 °C overnight in DUNN buffer. Membranes were stained in a 20% 2-propanol - 10% acetic acid - 0.1% amido black solution, destained in 2 washes with a 20% 2-propanol - 10% acetic acid solution, rinsed in distilled water, blocked for 30 minutes in PBS - 3% low fat milk, rinsed in PBS and incubated with primary antibodies (anti-activated JNK: Promega, V7931; anti-pan-JNK: R&D Systems, AF1387; anti-beta Actin: Pierce MA5- 15739; anti-GAPDH: Pierce, MA5-15738; anti-SQSTM1/p62 Antibody: Cell Signaling Technology 5114) diluted in PBS - 1% low fat milk at 4 °C overnight. After extensive washing with PBS, membranes were incubated for 2 hours in the presence of the HRP-conjugated secondary antibody, rinsed in PBS and HRP activity was detected using Millipore ECL according to the manufacturer recommendations. An Odyssey device (LI-COR Biosciences, Lincoln, Nebraska) was used to image the membranes.

Densitometric analysis of the western blots was performed using the Image Studio software (Li-COR Biosciences). Intensity of the pJNK signal was normalised to the intensity of the loading controls.

### ROS quantification

ROS detection was performed using the carboxy-H_2_DCFDA probe (Image-iT TM LIVE Green Reactive Oxygen Species Detection Kit (I36007), Molecular Probes Invitrogen. The apical tips of five coral branches were incubated in 750 μL of 0.45 μm filtered seawater containing 25 μmol.L^−1^ of carboxy-H_2_DCFDA probe in the presence of 0.001% DMSO or 10 μmol.L^−1^ SP600125 per condition. The apical tips were incubated for 30 minutes in the dark at 25 °C prior to exposure to the following conditions: 25 °C without UVR, 25 °C with UVR, 30 °C without UVR and 30 °C with UVR. Samples were then rinsed in filtered seawater and fluorescence was directly measured in the coral tissue with a Xenius SOF spectrofluorometer (SAFAS, Monaco) equipped with an optical fiber. The emission spectrum was measured between 515 and 550 nm with an excitation wavelength of 495 nm.

Human fibroblasts (0.5 × 10^6^ in 35 mm dishes) were incubated for 30 minutes in culture medium containing 25 μmol.L^−1^ of carboxy-H_2_DCFDA probe in the presence of 0.001% DMSO or 10 μmol.L^−1^ SP600125 prior to exposure to the following conditions25 °C without UVR, 25 °C with UVR, 30 °C without UVR and 30 °C with UVR for 10 minutes. Cells were imaged using a LEICA DM4000 microscope with a 40x objective and a Zyla5.5 sCMOS camera (Andor Technology Ltd, Belfast, Northern Ireland). The software ImageJ^77^ was used to quantify the fluorescence of at least five fields from three independent experiments per condition.

### Measurement of bleaching and cellular viability

Coral nubbins were treated for 1 hour with 5 μmol.L^−1^ SP600125 or 0.005% DMSO in standard culture conditions prior to exposure to UVR at 30 °C for 4 hours. Nubbins were then, rinsed, and placed back into control culture conditions for 24 hours. The symbiont content was measured as previously described^78^.

BJ-EHLT cells were seeded at a density of 10^6^ cells in 60 mm diameter dishes the day before the experiment. The cells were then treated for 30 minutes with 1 μmol.L^−0^f SP600125 or with 0.001% DMSO in standard culture conditions, exposed to UVR for 10 minutes and washed 3 times with culture medium before being placed back in normal culture conditions. The cell viability was assessed 24 hours after the initial treatment using an ADAM automatic fluorescence cell counter (AlphaMetrix Biotech, Rödermark, Germany) following the manufacturer’s recommendations.

### Statistical analysis

For ROS and western blot quantifications, conditions were compared using a three-way Analysis of Variance (ANOVA) with drugs (±SP 6000125), UVR (±UVR) and temperature (high or low temperature) as factors. The normality of the residuals and the homoscedasticity of the variances were tested using the Shapiro test and the Bartlett test, respectively. A Tukey’s Honest Significant Difference post hoc test was performed when significant differences were found. Fibroblast viability and the relative amounts of symbionts left at the end of the experiment were compared using a t-test. Normality of data was checked prior to running tests. For each test, we used 0.05 as alpha level. For western blot quantifications, the ANOVAs were ran on n = 3 replicates; and on n = 15 and n = 5 replicates per condition for ROS concentration in fibroblast and coral respectively. T-tests were performed on n = 3 and n = 4 replicates respectively.

## Additional Information

**How to cite this article:** Courtial, L. *et al*. The c-Jun N-terminal kinase prevents oxidative stress induced by UV and thermal stresses in corals and human cells. *Sci. Rep.* **7**, 45713; doi: 10.1038/srep45713 (2017).

**Publisher's note:** Springer Nature remains neutral with regard to jurisdictional claims in published maps and institutional affiliations.

## Supplementary Material

Supplementary Information

## References

[b1] Meyers L. A. & Bull J. J. Fighting change with change: Adaptive variation in an uncertain world. Trends Ecol. Evol. 17, 551–557 (2002).

[b2] Hofmann G. E. & Todgham A. E. Living in the now: physiological mechanisms to tolerate a rapidly changing environment. Annu. Rev. Physiol. 72, 127–145 (2010). 20148670 10.1146/annurev-physiol-021909-135900

[b3] Gallopin G. C. Linkages between vulnerability, resilience, and adaptive capacity. Glob. Environ. Chang. 16, 293–303 (2006).

[b4] Runchel C., Matsuzawa A. & Ichijo H. Mitogen-activated protein kinases in mammalian oxidative stress responses. Antioxid. Redox Signal. 15, 205–218 (2011). 21050144 10.1089/ars.2010.3733

[b5] Dhillon A. S., Hagan S., Rath O. & Kolch W. MAP kinase signalling pathways in cancer. Oncogene 26, 3279–90 (2007). 17496922 10.1038/sj.onc.1210421

[b6] Widmann C., Gibson S., Jarpe M. B. & Johnson G. L. Mitogen-activated protein kinase: conservation of a three-kinase module from yeast to human. Physiol. Rev. 79, 143–180 (1999). 9922370 10.1152/physrev.1999.79.1.143

[b7] Chen Z. . MAP Kinases. Chem. Rev. 101, 2449–2476 (2001). 11749383 10.1021/cr000241p

[b8] Dérijard B. . JNK1: A protein kinase stimulated by UV light and Ha-Ras that binds and phosphorylates the c-Jun activation domain. Cell 76, 1025–1037 (1994). 8137421 10.1016/0092-8674(94)90380-8

[b9] Bridge D. . FoxO and stress responses in the cnidarian Hydra vulgaris. PLoS One 5 (2010). 10.1371/journal.pone.0011686 PMC2908143 20657733

[b10] Garcia-Gomez C. . Cell survival after UV radiation stress in the unicellular chlorophyte Dunaliella tertiolecta is mediated by DNA repair and MAPK phosphorylation. J. Exp. Bot. 63, 5259–5274 (2012). 22859678 10.1093/jxb/ers185 PMC3430997

[b11] Parages M. L., Heinrich S., Wiencke C. & Jiménez C. Rapid phosphorylation of MAP kinase-like proteins in two species of Arctic kelps in response to temperature and UV radiation stress. Environ. Exp. Bot. 91, 30–37 (2013).

[b12] Yao C.-L. & Somero G. N. The impact of acute temperature stress on hemocytes of invasive and native mussels (Mytilus galloprovincialis and Mytilus californianus): DNA damage, membrane integrity, apoptosis and signaling pathways. J. Exp. Biol. 215, 4267–77 (2012). 22972891 10.1242/jeb.073577

[b13] Brunet A., . Stress-dependent regulation of FOXO transcription factors by the SIRT1 deacetylase. Science 303, 2011–2015 (2004). 14976264 10.1126/science.1094637

[b14] Shen H. M. & Liu Z. G. JNK signaling pathway is a key modulator in cell death mediated by reactive oxygen and nitrogen species. Free Radic. Biol. Med. 40, 928–939 (2006). 16540388 10.1016/j.freeradbiomed.2005.10.056

[b15] Davis R. J. Signal transduction by the JNK group of MAP kinases. Cell 103, 239–252 (2000). 11057897 10.1016/s0092-8674(00)00116-1

[b16] Liu J. & Lin A. Role of JNK activation in apoptosis: a double-edged sword. Cell Res. 15, 36–42 (2005). 15686625 10.1038/sj.cr.7290262

[b17] Tournier C. The 2 faces of JNK signaling in cancer. Genes Cancer 4, 397–400 (2013). 24349637 10.1177/1947601913486349 PMC3863340

[b18] Bachelor M. A. & Bowden G. T. UVA-mediated activation of signaling pathways involved in skin tumor promotion and progression. Semin. Cancer Biol. 14, 131–138 (2004). 15018897 10.1016/j.semcancer.2003.09.017

[b19] Kim E. K. & Choi E.-J. Pathological roles of MAPK signaling pathways in human diseases. Biochim. Biophys. Acta - Mol. Basis Dis. 1802, 396–405 (2010). 10.1016/j.bbadis.2009.12.009 20079433

[b20] Dent P., Yacoub A., Fisher P. B., Hagan M. P. & Grant S. MAPK pathways in radiation responses. Oncogene 22, 5885–96 (2003). 12947395 10.1038/sj.onc.1206701

[b21] Hoegh-Guldberg O. . Coral reefs under rapid climate change and ocean acidification. Science 318, 1737–1742 (2007). 18079392 10.1126/science.1152509

[b22] Doney S. C. . Climate change impacts on marine ecosystems. Ann. Rev. Mar. Sci. 4, 11–37 (2012). 10.1146/annurev-marine-041911-111611 22457967

[b23] D’Croz L & Maté J. L. The role of water temperature and UV radiation in the recovery of the experimentally bleached coral Pocillopora damicornis from the eastern Pacific Ocean (Panama). In Proceedings of the Ninth International Coral Reef Symposium, Bali, 23-27 October 2000 2, 1111–1116 (2002).

[b24] Drohan A. F., Thoney D. A. & Baker A. C. Synergistic effect of high temperature and ultraviolet-B radiation on the gorgonian *Eunicea tourneforti* (Octocorallia: Alcyonacea: Plexauridae). Bull. Mar. Sci. 77, 257–266 (2005).

[b25] Ferrier-Pagès C. . Effects of temperature and UV radiation increases on the photosynthetic efficiency in four scleractinian coral species. Biol. Bull. 213, 76–87 (2007). 17679722 10.2307/25066620

[b26] Yakovleva I., Bhagooli R., Takemura A. & Hidaka M. Differential susceptibility to oxidative stress of two scleractinian corals: Antioxidant functioning of mycosporine-glycine. Comp. Biochem. Physiol. - B Biochem. Mol. Biol. 139, 721–730 (2004). 15581804 10.1016/j.cbpc.2004.08.016

[b27] Banaszak A. T. & Lesser M. P. Effects of solar ultraviolet radiation on coral reef organisms. Photochem. Photobiol. Sci. 8, 1276 (2009). 19707616 10.1039/b902763g

[b28] Lesser M. P. Oxidative stress in marine environments: biochemistry and physiological ecology. Annu. Rev. Physiol. 68, 253–278 (2006). 16460273 10.1146/annurev.physiol.68.040104.110001

[b29] Siboni N. . Using bacterial extract along with differential gene expression in Acropora millepora Larvae to decouple the processes of attachment and metamorphosis. PLoS One 7 (2012). 10.1371/journal.pone.0037774 PMC3359992 22655067

[b30] Sun J., Chen Q., Lun J. C. Y., Xu J. & Qiu J. W. PcarnBase: Development of a transcriptomic database for the brain coral Platygyra carnosus. Mar. Biotechnol. 15, 244–251 (2013). 22875536 10.1007/s10126-012-9482-z

[b31] Mayfield A. B., Hsiao Y. Y., Fan T. Y., Chen C. S. & Gates R. D. Evaluating the temporal stability of stress-activated protein kinase and cytoskeleton gene expression in the Pacific reef corals Pocillopora damicornis and Seriatopora hystrix. J. Exp. Mar. Bio. Ecol. 395, 215–222 (2010).

[b32] Miller D. J. . The innate immune repertoire in cnidaria-ancestral complexity and stochastic gene loss. Genome Biol. 8, R59 (2007). 17437634 10.1186/gb-2007-8-4-r59 PMC1896004

[b33] Shinzato C. . Using the Acropora digitifera genome to understand coral responses to environmental change. Nature 476, 320–323 (2011). 21785439 10.1038/nature10249

[b34] van de Water J. A. J. M., Lamb J. B., van Oppen M. J. H., Willis B. L. & Bourne D. G. Comparative immune responses of corals to stressors associated with offshore reef-based tourist platforms. Conserv. Physiol. 3, cov032 (2015). 27293717 10.1093/conphys/cov032 PMC4778433

[b35] Daly M. . The phylum Cnidaria: A review of phylogenetic patterns and diversity 300 years after Linnaeus. Zootaxa 182, 127–182 (2007).

[b36] van de Water J. A. J. M. . Elevated seawater temperatures have a limited impact on the coral immune response following physical damage. Hydrobiologia 759, 201–214 (2015).

[b37] van De Water J. A. J. M. . The coral immune response facilitates protection against microbes during tissue regeneration. Mol. Ecol. 24, 3390–3404 (2015). 26095670 10.1111/mec.13257

[b38] Kaminska B. MAPK signalling pathways as molecular targets for anti-inflammatory therapy - From molecular mechanisms to therapeutic benefits. Biochim. Biophys. Acta - Proteins Proteomics 1754, 253–262 (2005). 10.1016/j.bbapap.2005.08.017 16198162

[b39] Liew Y. J. . Identification of microRNAs in the coral Stylophora pistillata. PLoS One 9, 1–11 (2014). 10.1371/journal.pone.0091101 PMC3962355 24658574

[b40] Shinzato C., Mungpakdee S., Satoh N. & Shoguchi E. A genomic approach to coral-dinoflagellate symbiosis: Studies of Acropora digitifera and Symbiodinium minutum. Front. Microbiol. 5, 1–17 (2014). 25071748 10.3389/fmicb.2014.00336 PMC4083563

[b41] Krishna S. . Deep sequencing reveals unique small RNA repertoire that is regulated during head regeneration in Hydra magnipapillata. Nucleic Acids Res. 41, 599–616 (2013). 23166307 10.1093/nar/gks1020 PMC3592418

[b42] Barshis D. J., Ladner J. T., Oliver T. A. & Palumbi S. R. Lineage-specific transcriptional profiles of Symbiodinium spp. unaltered by heat stress in a coral host. Mol. Biol. Evol. 31, 1343–1352 (2014). 24651035 10.1093/molbev/msu107

[b43] Hanks S. K. & Hunter T. Protein kinases 6. The eukaryotic protein kinase superfamily: kinase (catalytic) domain structure and classification. FASEB J. 9, 576–596 (1995). 7768349

[b44] Ip Y. T. & Davis R. J. Signal transduction by the c-Jun N-terminal kinase (JNK)—from inflammation to development. Curr. Opin. Cell Biol. 10, 205–219 (1998). 9561845 10.1016/s0955-0674(98)80143-9

[b45] Philipp I., Holstein T. W. & Hobmayer B. HvJNK, a Hydra member of the c-Jun NH2-terminal kinase gene family, is expressed during nematocyte differentiation. Gene Expr. Patterns 5, 397–402 (2005). 15661646 10.1016/j.modgep.2004.09.007

[b46] Manole S., Richards E. J & Meyer A. S. JNK pathway activation modulates acquired resistance to EGFR/HER2 targeted therapies. Cancer Res(2016). 10.1158/0008-5472.CAN-16-0123 PMC5026573 27450453

[b47] Sayeed A. . β1 integrin- and JNK-dependent tumor growth upon hypofractionated radiation. *Oncotarget* (2016). 10.18632/oncotarget.10522 PMC5288136 27438371

[b48] Bennett B. L. . SP600125, an anthrapyrazolone inhibitor of Jun N-terminal kinase. Proc. Natl. Acad. Sci. USA 98, 13681–13686 (2001). 11717429 10.1073/pnas.251194298 PMC61101

[b49] Putnam N. H. . Sea anemone genome reveals ancestral eumetazoan gene repertoire and genomic organization. Science 317, 86–94 (2007). 17615350 10.1126/science.1139158

[b50] Philipp I., Holstein T. W. & Hobmayer B. HvJNK, a Hydra member of the c-Jun NH 2-terminal kinase gene family, is expressed during nematocyte differentiation. Gene Expr. patterns 5, 397–402 (2005). 15661646 10.1016/j.modgep.2004.09.007

[b51] Davis R. In Inflammatory Processes(eds Letts L. G. & Morgan D.) 13–21 (Birkhäuser Basel, 2000).

[b52] Lesser M. P., Stochaj W. R., Tapley D. W. & Shick J. M. Coral Reefs effects of irradiance, ultraviolet radiation, and temperature. Coral Reefs 8, 225–232 (1990).

[b53] D’Croz L., Maté J. L. & Oke J. E. Responses to elevated sea water temperature and UV radiation in the coral Porites lobata from upwelling and non-upwelling environments on the Pacific coast of Panama. Bull. Mar. Sci. 69, 203–214 (2001).

[b54] Shick J., Lesser M. P. & Jokiel P. L. Effects of ultraviolet radiation on corals and other coral reef organisms. Glob. Chang. Biol. 2, 527–545 (1996).

[b55] Hoegh-Guldberg O. Climate change, coral bleaching and the future of the world’s coral reefs. Mar. Freshw. Res. 50, 839 (1999).

[b56] Chaitanya G. V., Steven A. J. & Babu P. P. PARP-1 cleavage fragments: signatures of cell-death proteases in neurodegeneration. Cell Commun Signal 8, 31 (2010). 21176168 10.1186/1478-811X-8-31 PMC3022541

[b57] Bernerd F. & Asselineau D. An organotypic model of skin to study photodamage and photoprotection *in vitro*. J. Am. Acad. Dermatol. 58, S155–S159 (2008). 18410802 10.1016/j.jaad.2007.08.050

[b58] Marionnet C. . Different oxidative stress response in keratinocytes and fibroblasts of reconstructed skin exposed to non extreme daily-ultraviolet radiation. PLoS One 5, e12059 (2010). 20706594 10.1371/journal.pone.0012059 PMC2919404

[b59] Tournier C. . Requirement of JNK for Stress- Induced Activation of the Cytochrome c-Mediated Death Pathway. Science 288, 870–874 (2000). 10797012 10.1126/science.288.5467.870

[b60] Yabu T. . Stress-induced ceramide generation and apoptosis via the phosphorylation and activation of nSMase1 by JNK signaling. Cell Death Differ. 22, 258–73 (2015). 25168245 10.1038/cdd.2014.128 PMC4291487

[b61] Cao G. . EGCG protects against UVB-induced apoptosis via oxidative stress and the JNK1/c-Jun pathway in ARPE19 cells. Mol. Med. Rep. 5, 54–59 (2012). 21909619 10.3892/mmr.2011.582

[b62] Khoshnood B., Dacklin I. & Grabbe C. Urm1: an essential regulator of JNK signaling and oxidative stress in Drosophila melanogaster. Cell. Mol. Life Sci. 73, 1939–1954 (2016). 26715182 10.1007/s00018-015-2121-x PMC11108535

[b63] Wang M. C., Bohmann D. & Jasper H. JNK signaling confers tolerance to oxidative stress and extends lifespan in Drosophila. Dev. Cell 5, 811–816 (2003). 14602080 10.1016/s1534-5807(03)00323-x

[b64] Essers M. A. G. . FOXO transcription factor activation by oxidative stress mediated by the small GTPase Ral and JNK. EMBO J. 23, 4802–12 (2004). 15538382 10.1038/sj.emboj.7600476 PMC535088

[b65] Venugopal R. & Jaiswal a. K. Nrf2 and Nrf1 in association with Jun proteins regulate antioxidant response element-mediated expression and coordinated induction of genes encoding detoxifying enzymes. Oncogene 17, 3145–3156 (1998). 9872330 10.1038/sj.onc.1202237

[b66] Kops G. J. P. L. . Forkhead transcription factor FOXO3a protects quiescent cells from oxidative stress. Nature 419, 316–321 (2002). 12239572 10.1038/nature01036

[b67] Brown M. . An initial phase of JNK activation inhibits cell death early in the endoplasmic reticulum stress response. J. Cell Sci. 2, jcs.179127 (2016). 10.1242/jcs.179127 PMC5172423 27122189

[b68] Flot J.-F. . Incongruence between morphotypes and genetically delimited species in the coral genus Stylophora: phenotypic plasticity, morphological convergence, morphological stasis or interspecific hybridization? BMC Ecol. 11, 1 (2011). 21970706 10.1186/1472-6785-11-22 PMC3269986

[b69] Biroccio A. . TRF2 inhibits a cell-extrinsic pathway through which natural killer cells eliminate cancer cells. Nat. Cell Biol. 15, 818–828 (2013). 23792691 10.1038/ncb2774

[b70] Iordanov M. S. . Ribotoxic stress response: activation of the stress-activated protein kinase JNK1 by inhibitors of the peptidyl transferase reaction and by sequence-specific RNA damage to the alpha-sarcin/ricin loop in the 28S rRNA. Mol. Cell. Biol. 17, 3373–3381 (1997). 9154836 10.1128/mcb.17.6.3373 PMC232190

[b71] Al-Moghrabi S., Goiran C., Allemand D., Speziale N. & Jaubert J. Inorganic carbon uptake for photosynthesis by the symbiotic coral-dinoflagellate association.2. Mechanisms for bicarbonate uptake. J. Exp. Mar. Bio. Ecol. 199, 227–248 (1996).

[b72] Courtial L., Roberty S., Shick J. M., Houlbrèque F. & Ferrier-Pagès C. Interactive effects of ultraviolet radiation and thermal stress on two reef-building corals. Limnol Oceanogr(2017).

[b73] Shick J. M. The continuity and intensity of ultraviolet irradiation affect the kinetics of biosynthesis, accumulation, and conversion of mycosporine-like amino acids (MAAs) in the coral Stylophora pistillata. Limnol. Oceanogr. 49, 442–458 (2004).

[b74] Torres-Pérez J. L. & Armstrong R. A. Effects of UV radiation on the growth, photosynthetic and photoprotective components, and reproduction of the Caribbean shallow-water coral Porites furcata. Coral Reefs 31, 1077–1091 (2012).

[b75] Shick J. M., Lioud S., Reynaud S., Ferrier-Pagès C. & Gattuso J.-P. Ultraviolet-B radiation stimulates shikimate pathway-dependent accumulation of mycosporine-like amino acids in the coral Stylophora pistillata despite decreases in its population of symbiotic dinoflagellates. Limnol. Oceanogr. 44, 1667–1682 (1999).

[b76] Laemmli U. K. Cleavage of structural proteins during the assembly of the head of bacteriophage T4. Nature 227, 680–685 (1970). 5432063 10.1038/227680a0

[b77] Schneider C., Rasband W. S. & Eliceiri K. W. NIH Image to ImageJ: 25 years of image analysis. Nat. Methods 9, 671–675 (2012). 22930834 10.1038/nmeth.2089 PMC5554542

[b78] Godinot C., Ferrier-Pagès C., Sikorski S. & Grover R. Alkaline phosphatase activity of reef-building corals. Limnol. Oceanogr. 58, 227–234 (2013).

