# Supplementary material for: The c-Jun N-terminal kinase prevents oxidative stress induced by UV and thermal stresses in corals and human cells
Source: Sci Rep. 2017 Apr 4;7:45713. doi: 10.1038/srep45713 (PMC5379690; doi:10.1038/srep45713)
Supplement: Supplementary Information [file srep45713-s1.pdf]

# Supplementary information

The c-Jun N-terminal kinase prevents oxidative stress induced by UV and thermal stresses in corals and human cells

L.Courtial, V. Picco, R. Grover, Y.Cormerais, C. Rottier, A. Labbe, G. Pagès and C. Ferrier-Pagès

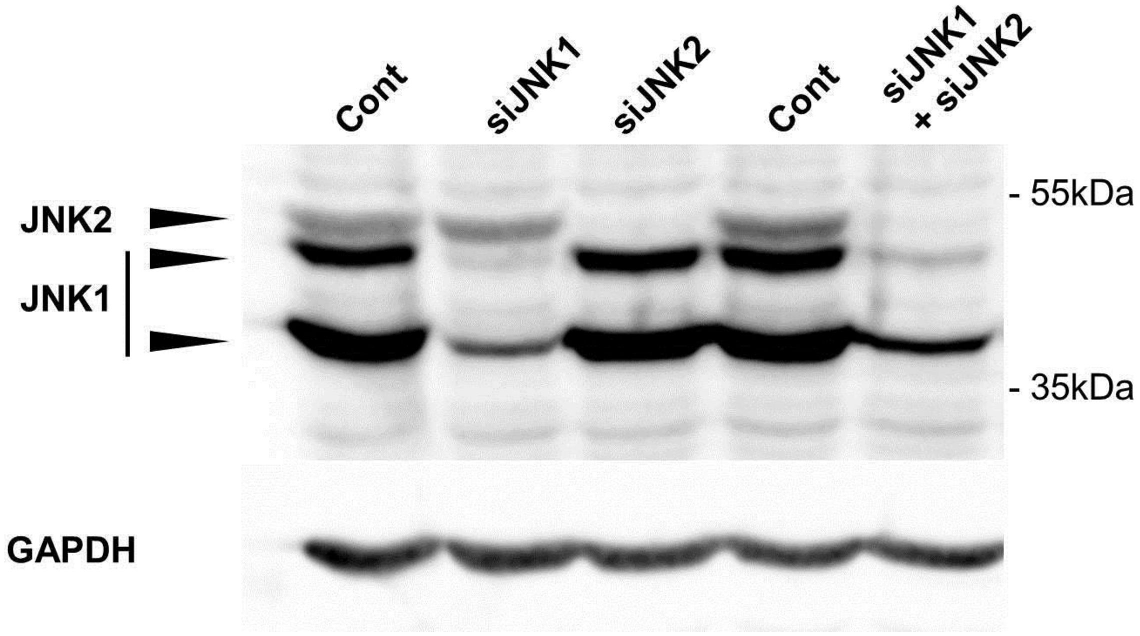


**Supplementary Figure S1: JNK genes and isoforms expression in human BJ fibroblasts.** The determination of the genes and isoforms expression was conducted by a siRNA-based approach. Briefly, siRNA pools directed against the human JNK1 and JNK2 genes (respectively ON-TARGETplus Human MAPK8 (5599) and MAPK9 (5601) SMARTpools) and the non-target control siRNA pool (the ON-TARGETplus Non-targeting Pool) were transfected with Dharmafect 1 transfection reagent (T-2001-02) as recommended by the manufacturer (GE Healthcare Europe, Velizy-Villacoublay, France). Cell lysates prepared for western blotting as described in the manuscript and were analyzed by immunoblot using a pan-JNK antibody. In the figure, the Cont, siJNK1, siJNK2 and siJNK1+2 columns represent cells transfected with non-target siRNAs, with siRNAs directed against JNK1 (siJNK1), JNK2 (siJNK2) or a combination of both siRNAs (siJNK1+siJNK2) respectively. GAPDH served as loading control. Molecular weight standards are indicated on the right part of the figure.

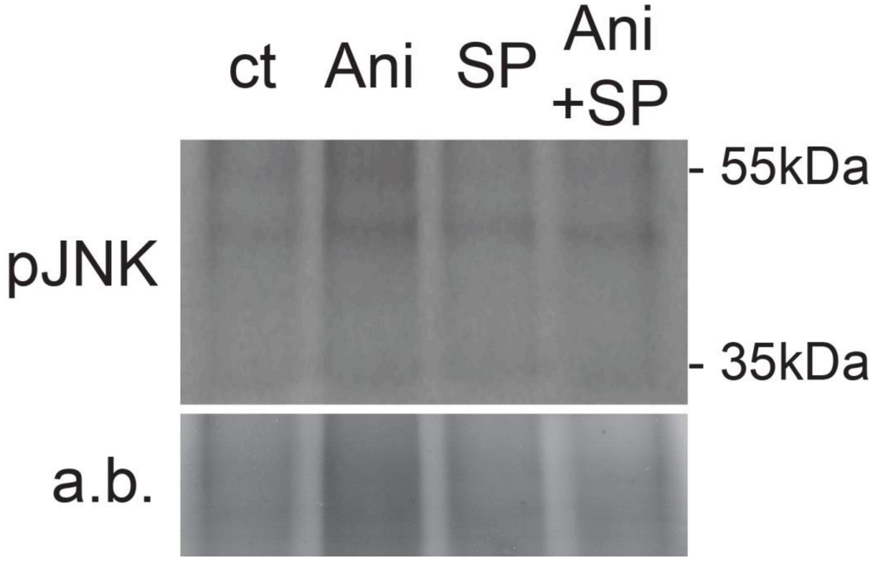


**Supplementary Figure S2: Absence of JNK activity in Symbiodinium clade B1.** Immunoblot confirming the absence of activated JNK (pJNK) in Symbiodinium clade B1 cultures treated as described for coral nubbins, i.e. in the absence or presence of SP600125 (SP) and anisomycin (Ani and Ani+SP). Amido black staining (a.b.) served as loading control.

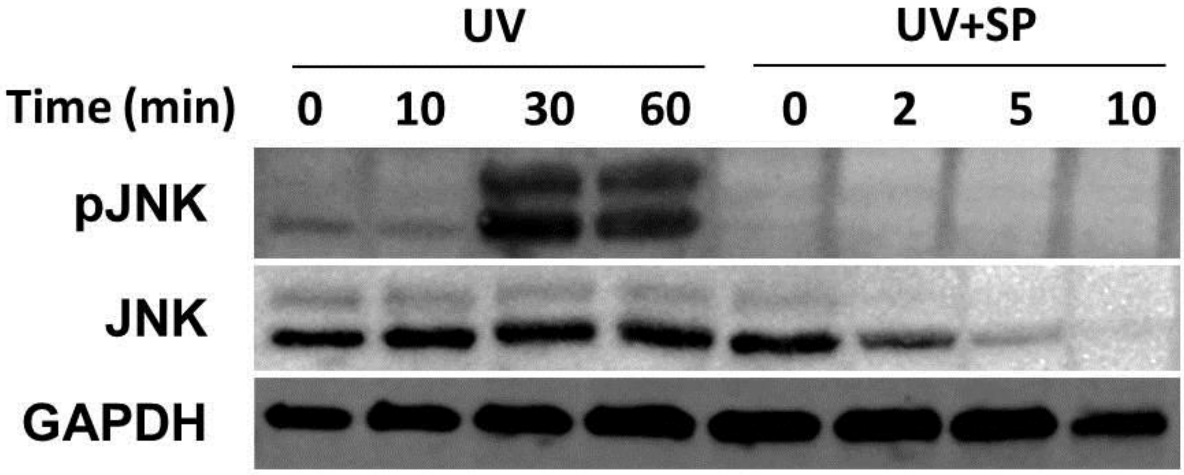


**Supplementary Figure S3: Time course assessment of JNK activation and degradation.** Immunoblots revealing activated JNK (pJNK) in human fibroblasts exposed to UV radiation for the indicated times in the absence (UV) or presence (UV+SP) of JNK inhibitor SP600125. JNK and GAPDH served as loading control.

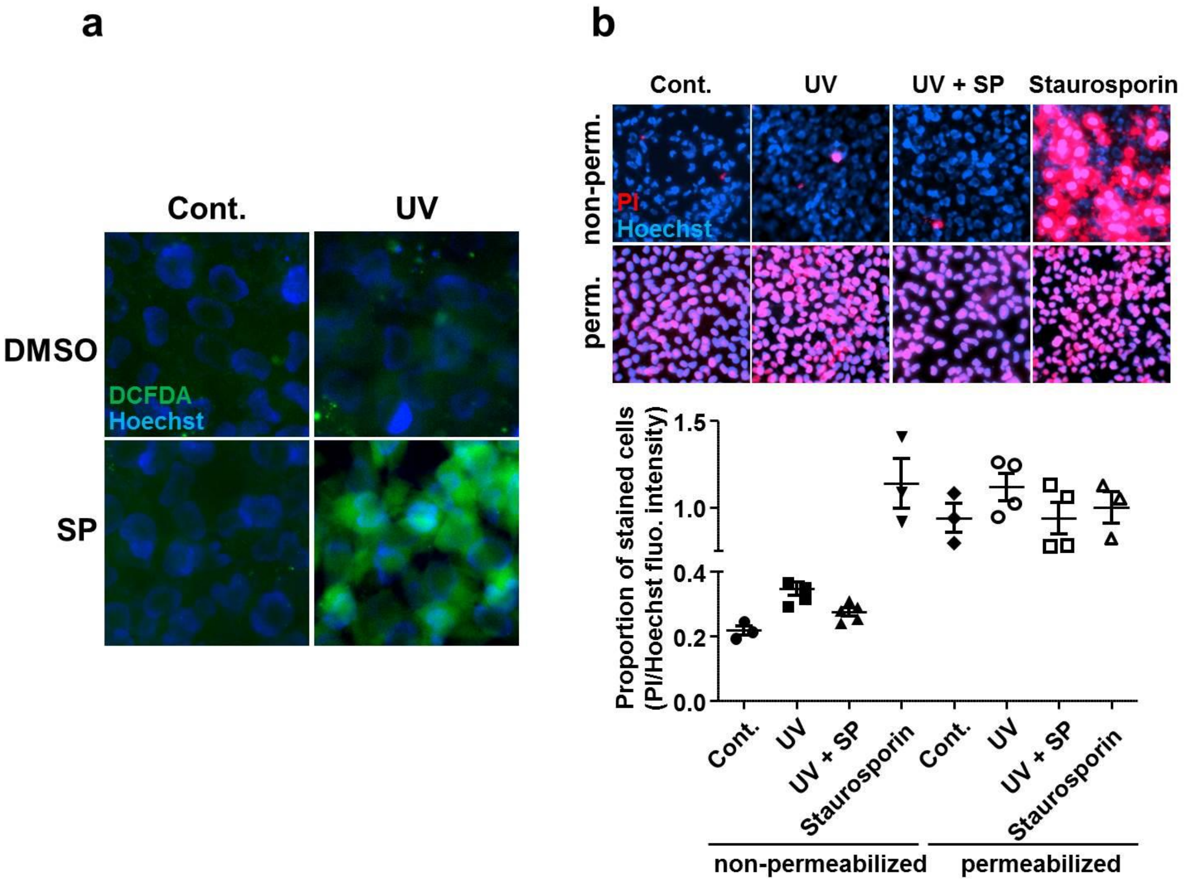


**Supplementary Figure S4: Assessment of cell membrane permeability by dual Hoechst 33342 and propidium iodide staining after exposure to UVR and JNK pharmacological inhibitor.**

Cells were treated with UVR and/or SP600125 as previously described and incubated for 20 minutes with 30 µM propidium iodide and 1µg.mL$^{-1}$ Hoechst 33342. Hoechst 33342 is a cell membrane-permeant DNA dye with excitation/emission maxima of 361/497 nm whereas propidium iodide is a cell membrane-impermeable DNA dye with excitation/emission maxima of 535⁄617 nm. The difference in cell-permeability between the two dyes allows a discrimination of the cells with damaged membranes from intact cells. Cells were imaged using a Leica DMI4000 microscope as previously described. In the figure, (a) Cells stained with the ROS carboxy-DCFDA probe (DCFDA, green) still retained fluorescence 30 minutes after exposure to a combination of UVR and SP600125. (b) Intracellular staining of the cells with the cell impermeable propidium iodide dye (PI, red) did not increase under UVR and SP600125 single or combined treatments, as opposed to treatment with the apoptosis inducer staurosporin or the permeabilizing agent Triton X100 (perm.)

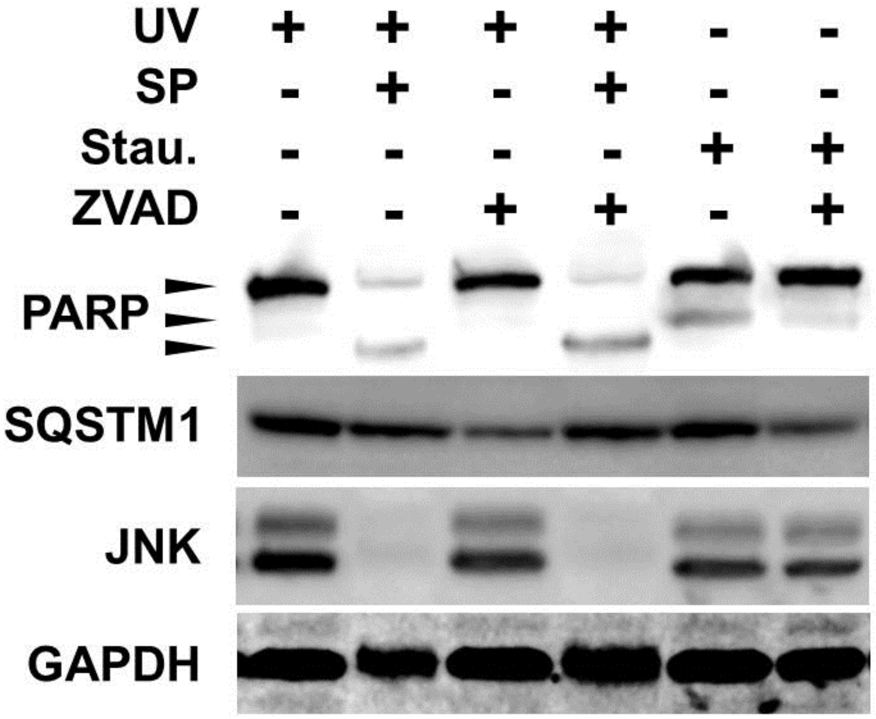


**Figure S5: Activation of cell death pathways in various experimental conditions.** Immunoblots revealing PARP, SQSTM1 and JNK in human fibroblasts in the absence (-) or presence (+) of UV radiation (UV), the JNK inhibitor SP600125 (SP), the pharmacological activator of apoptosis staurosporin (Stau.) and the caspase inhibitor Z-VAD-FMK (ZVAD). GAPDH served as loading control.

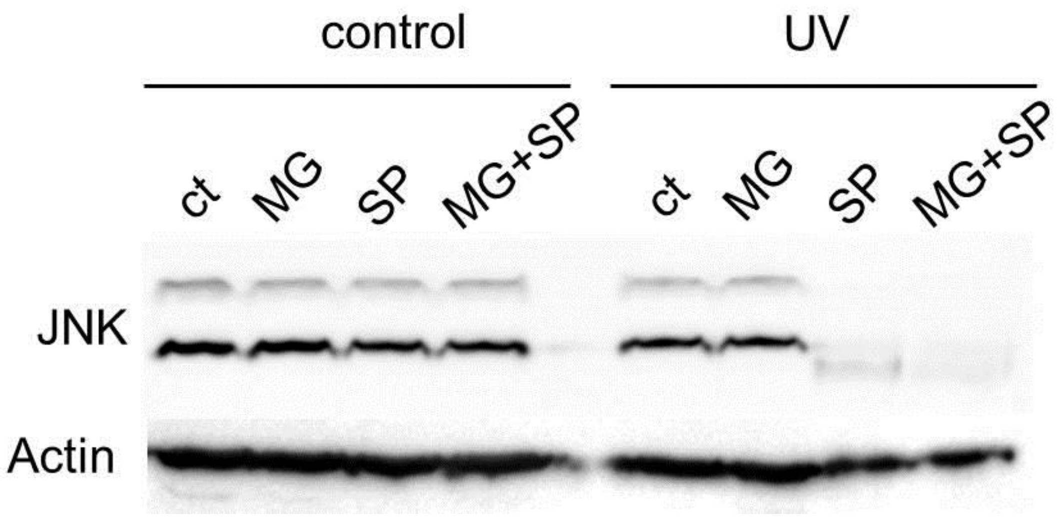


**Figure S6: Pharmacological inhibition of the proteasome does not prevent JNK degradation.** Immunoblot revealing total JNK form (JNK) in human fibroblasts in the absence (ct) or presence of the proteasome inhibitor MG132 (MG), the JNK inhibitor SP600125 (SP) or a combination of both (MG+SP) and exposed (UV) or not (control) to UVR. Actin served as loading control.

| | | Human fibroblasts | | | *S. pistillata* | | |
|---|---|---|---|---|---|---|---|
| | **DF** | **Mean Sq** | **F value** | **P** | **Mean Sq** | **F value** | **P** |
| **Temperature** | 1 | 56.88 | 15.29 | **$1.25x10^{-3}$** | 11.01 | 9.44 | **$5.38x10^{-3}$** |
| **UVR** | 1 | 1.92 | 0.52 | 0.48 | 15.36 | 13.18 | **$1.40x10^{-3}$** |
| **Inhibitor** | 1 | 106.13 | 28.53 | **$6.62x10^{-5}$** | 27.60 | 23.68 | **$6.50x10^{-5}$** |
| **Temperature:UVR** | 1 | 0.84 | 0.23 | 0.64 | 0.15 | 0.13 | 0.72 |
| **Temperature:inhibitor** | 1 | 72.99 | 19.62 | **$4.20x10^{-4}$** | 0.00 | 0.00 | 0.96 |
| **UVR:inhibitor** | 1 | 0.19 | 0.05 | 0.83 | 0.04 | 0.04 | 0.58 |
| **Temperature:UVR:inhibitor** | 1 | 1.10 | 0.30 | 059 | 0.13 | 0.15 | 0.70 |

**Supplementary table 1: Results of the three-way ANOVAs testing the difference between temperature, UVR and SP600125 effects on JUNK expression.** ANOVAs were performed on n=4 and 0.05 was used as the alpha level. Statistically significant results are highlighted in red.

| | | Human fibroblasts | | | *S. pistillata* | | |
|---|---|---|---|---|---|---|---|
| | DF | Mean Sq | F value | P | Mean Sq | F value | P |
| **Temperature** | 1 | 0.19 | 12.73 | **$5.73x10^{-4}$** | 5.70 | 42.30 | **$2.55x10^{-7}$** |
| **UVR** | 1 | 22.08 | 1520.19 | **$<2x10^{-16}$** | 11.33 | 84.18 | **$1.78x10^{-10}$** |
| **Inhibitor** | 1 | 29.78 | 2050.67 | **$<2x10^{-16}$** | 18.32 | 136.08 | **$4.60x10^{-13}$** |
| **Temperature:UVR** | 1 | 0.00 | 0.28 | 0.60 | 1.45 | 10.74 | **$2.53x10^{-3}$** |
| **Temperature:inhibitor** | 1 | 0.01 | 0.92 | 0.34 | 1.38 | 10.24 | **$3.10x10^{-3}$** |
| **UVR:inhibitor** | 1 | 9.55 | 657.59 | **$<2x10^{-16}$** | 0.00 | 0.00 | 0.99 |
| **Temperature:UVR:inhibitor** | 1 | 0.03 | 2.08 | 0.15 | 0.21 | 1.58 | 0.22 |

**Supplementary table 2: Results of the three-way ANOVAs testing the difference between temperature, UVR and SP600125 effects on the cellular ROS concentration.** ANOVAs were performed on n=15 for fibroblasts and n=5 for *S. pistillata*. 0.05 was used as the alpha level. Statistically significant results are highlighted in red.

| | | DF | t value | P |
|---|---|---|---|---|
| | **Ct+SP : UV-SP** | 2,47 | -0,91 | 0.44 |
| **Human fibroblasts** | **Ct+SP : UV+SP** | 3,99 | 12,20 | **$2,62x10^{-4}$** |
| | **UV+SP : UV-SP** | 2,51 | -17,58 | **$1,07x10^{-3}$** |
| | **Ct+SP : UVT-SP** | 6,79 | -0,70 | 0.51 |
| ***S. pistillata*** | **Ct+SP : UVT+SP** | 6,56 | 2,46 | **$4,59x10^{-2}$** |
| | **UVT+SP : UVT-SP** | 7,95 | 3,23 | **$1,21x10^{-2}$** |

**Supplementary table 3. Results of the T-tests testing the differences between conditions: control (Ct), UVR (UV), UVR and high temperature (UVT) with or without SP600125 (+/- SP).** T-tests were performed on n=3 and n=4 for fibroblasts and coral nubbins respectively. 0.05 was used as the alpha level.

# Full lenght blots

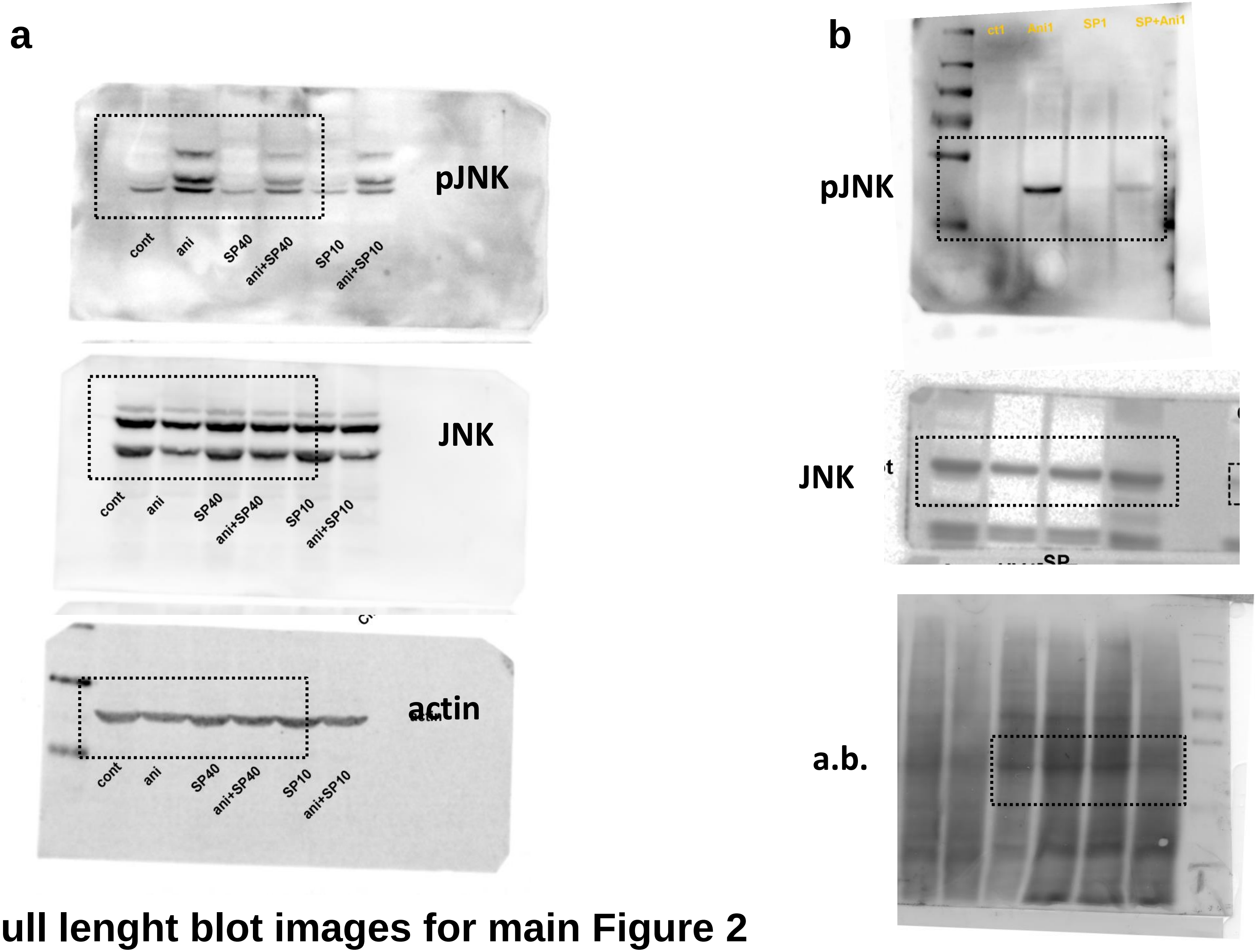


Full lenght blot images for main Figure 2

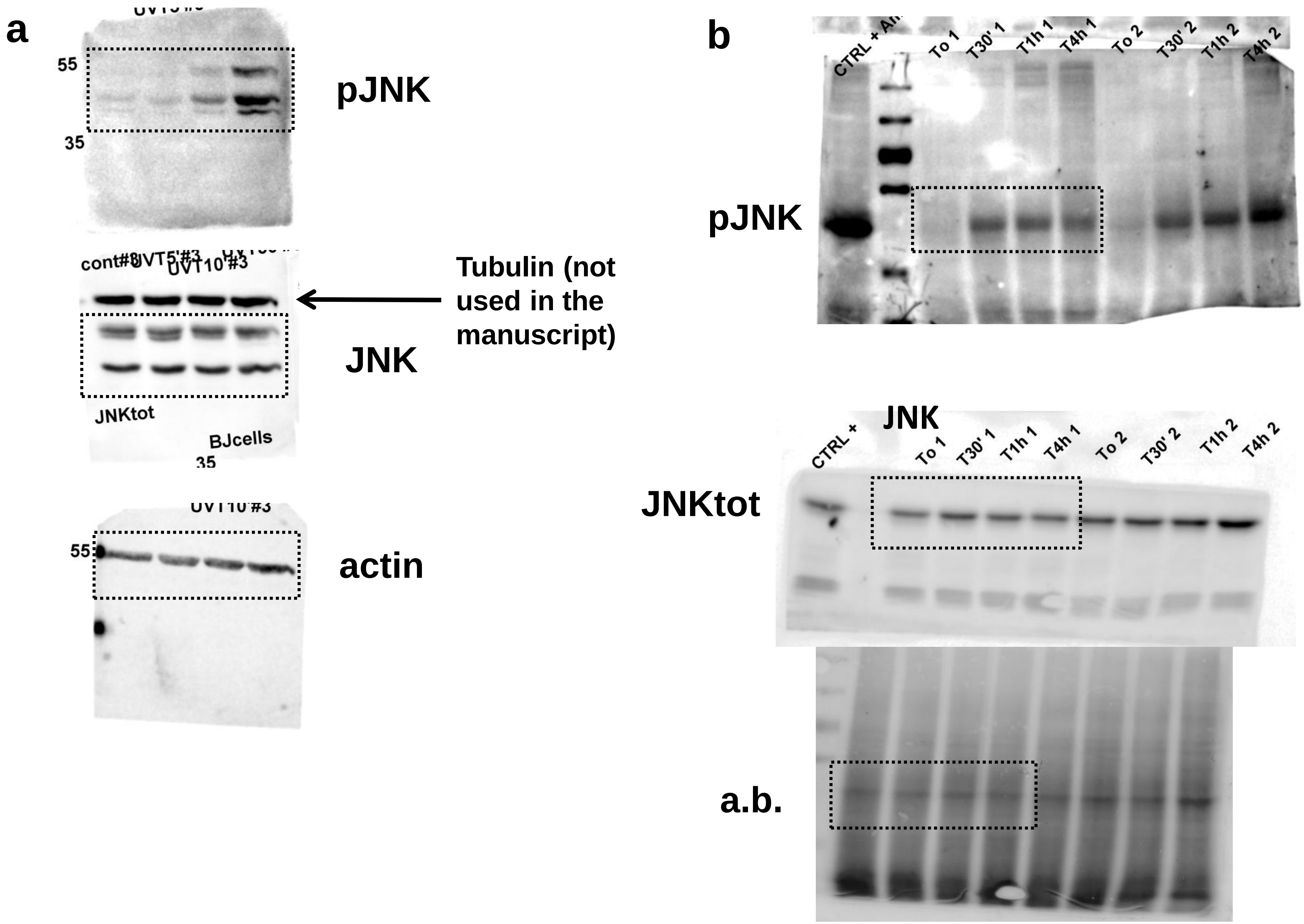


**Full lenght blot images for main Figure 3**

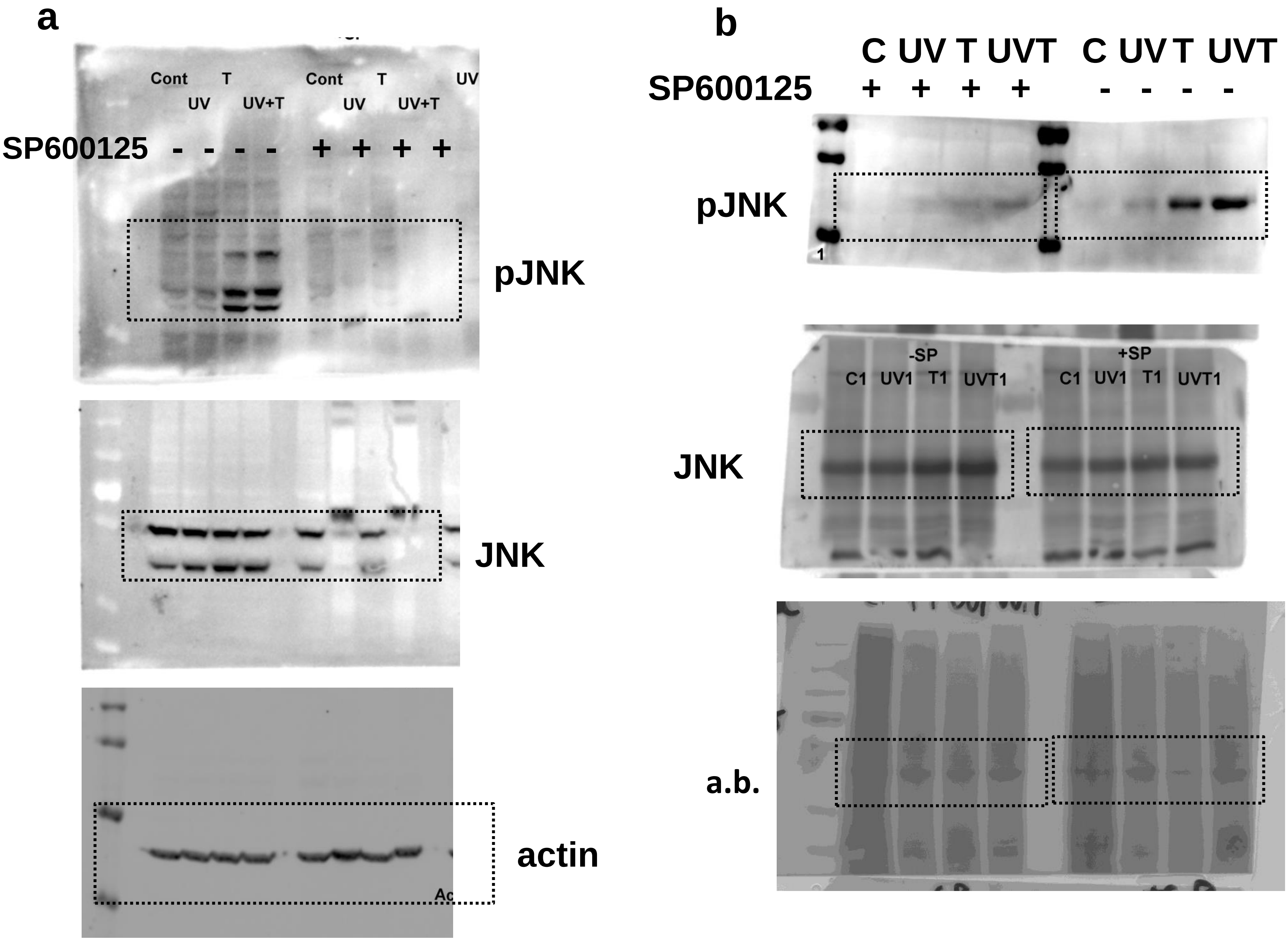


Full lenght blot images for main Figure 4
